# Exploring the Molecular Landscape of Myelofibrosis, with a Focus on Ras and Mitogen-Activated Protein (MAP) Kinase Signaling

**DOI:** 10.3390/cancers15184654

**Published:** 2023-09-21

**Authors:** Samuel B. Reynolds, Kristen Pettit, Malathi Kandarpa, Moshe Talpaz, Qing Li

**Affiliations:** 1 Division of Hematology/Oncology, Department of Medicine, University of Michigan, Ann Arbor, MI 48109, USA; krpettit@med.umich.edu (K.P.); mtalpaz@med.umich.edu (M.T.); 2 Department of Cell and Developmental Biology, University of Michigan, Ann Arbor, MI 48109, USA; malathi@med.umich.edu

**Keywords:** anemia, molecular, myelofibrosis, myeloproliferative, Ras, signaling, thrombocytopenia

## Abstract

**Simple Summary:**

Myelofibrosis is a disease arising from bone marrow driven by mutations in stem cells, which results in progressive failure of marrow production in red and white blood cells as well as platelets. Patients can also develop leukemia (a cancer of white blood cells) over time. While the only cure is a bone marrow transplant, many patients are not candidates and require treatment for symptomatic disease. The most common therapies target a protein known as “Jak” but resistance to these agents is common, making the exploration of alternative mechanisms for how such resistance manifests essential. Beyond Jak, another emerging yet under-explored target in myelofibrosis is a group of proteins known as “Ras/MAP Kinase”. The aim of this review is to present research into Ras/MAP Kinase signaling and its role in myelofibrosis, which we hope will expand how providers view and target this disease, ultimately improving the lives of patients.

**Abstract:**

Myelofibrosis (MF) is a clonal myeloproliferative neoplasm (MPN) characterized clinically by cytopenias, fatigue, and splenomegaly stemming from extramedullary hematopoiesis. MF commonly arises from mutations in *JAK2*, *MPL*, and *CALR*, which manifests as hyperactive Jak/Stat signaling. Triple-negative MF is diagnosed in the absence of *JAK2*, *MPL*, and *CALR* but when clinical, morphologic criteria are met and other mutation(s) is/are present, including *ASXL1*, *EZH2*, and *SRSF2*. While the clinical and classic molecular features of MF are well-established, emerging evidence indicates that additional mutations, specifically within the Ras/MAP Kinase signaling pathway, are present and may play important role in disease pathogenesis and treatment response. *KRAS* and *NRAS* mutations alone are reportedly present in up to 15 and 14% of patients with MF (respectively), and other mutations predicted to activate Ras signaling, such as *CBL*, *NF1*, *BRAF*, and *PTPN11*, collectively exist in as much as 21% of patients. Investigations into the prevalence of *RAS* and related pathway mutations in MF and the mechanisms by which they contribute to its pathogenesis are critical in better understanding this condition and ultimately in the identification of novel therapeutic targets.

## 1. Introduction

Myelofibrosis (MF) is a myeloproliferative neoplasm (MPN) arising from a clonal proliferation of hematopoietic stem cells. The clinical manifestations stemming from this proliferation in the bone marrow compartment include constitutional symptoms (fevers, chills, and weight loss), fatigue, and splenomegaly. Long-term sequelae may include leukemic transformation and/or severe anemia and thrombocytopenia in the setting of cytopenic disease [1]. MPNs including polycythemia vera (PV), essential thrombocythemia (ET), and MF are characterized by overactive Jak/Stat pathway signaling, usually resulting from mutations in *JAK2*, *MPL*, or *CALR*.

Management in myelofibrosis varies by clinical risk; lower-risk patients may be closely observed while high risk disease should be approached with consideration for allogeneic stem cell transplantation. Patients with bothersome splenomegaly and/or constitutional symptoms are generally treated with *JAK* pathway inhibitors, which include ruxolitinib, fedratinib, or for cytopenic disease, pacritinib [2,3]. However, the modest disease modification and survival changes in those treated with *JAK* inhibitors has been disappointing [4,5,6]. Lack of response or eventual resistance to *JAK* inhibitors is common, and mechanisms of resistance are incompletely understood.

Understanding the genetic and molecular landscape of MF will inform new and effective therapeutics. Emerging evidence indicates that MF has a diverse mutational profile and many mutated genes such as *ASXL1*, *EZH2*, *IDH1/2*, *SRSF2*, and *U2AF1* are associated with high-risk disease features. Their presence, in fact, confers valuable prognostic information that may be utilized in risk stratification models (e.g., Mutation-Enhanced International Prognostic Score System for Transplantation-Age Patients with Primary Myelofibrosis, MIPSSv2) [7]. Recent data suggest that mutations of the Ras signaling pathway are observed in MF and may be associated with high-risk features and poor outcomes [8]. This review will summarize studies delineating the genetic and molecular features of MF with a focus on mutations that activate Ras signaling and their potential implications in the prognosis and treatment of MF.

## 2. Driver Mutations in Myelofibrosis

The primary driver mutations in myelofibrosis are well-established and mainly include *JAK2*, *CALR*, and *MPL* mutations. Numerous studies have supported their presence as being essential in the diagnostic consideration of overt myelofibrosis, including the most recent International Consensus Classification (ICC) Guidelines [9]. Specifically, one of the three major criteria for overt MF diagnosis requires the presence of either a *CALR*, *JAK2*, or *MPL* mutation, an alternative marker of clonality (including *EZH2*, *ASXL1*, and *TET2*) or absence of reactive reticulin fibrosis in the bone marrow.

### 2.1. JAK2

**Figure 1:**
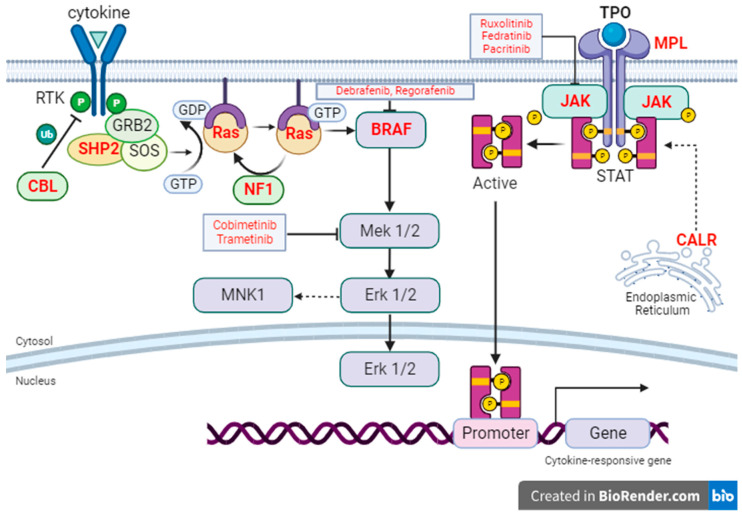
Intracellular signaling through Ras/MAP Kinase and Jak/Stat pathways. Genes whose mutations have been found in MF are labeled with red bold font. Within this model, loss of function in *NF1* and *CBL*; gain of function mutations in *PTPN11* (encoding SHP2); and activating mutations in *K*/*NRAS*, lead to hyperactive Ras signaling [35,36]. *JAK2* and *MPL* gain-of-function mutations result in hyperactive Jak/Stat signaling. Mutations in *CALR*, lastly, induce constitutive activation of Mpl and Jak/Stat signaling, particularly in myeloproliferative neoplasms [37]. Various small molecular inhibitors against targets in the Ras/MAPK pathway or JAK/Stat pathway are included in text boxes in red font [38].

Janus kinase 2 (*JAK2*) is a member of the family of Jak nonreceptor tyrosine kinases and is an essential component of normal hematopoiesis [10]. Activation of receptor tyrosine kinases by growth factors, cytokines, or chemokines results in phosphorylation and activation of Jak2, which leads to Stat phosphorylation and activation of downstream targets of Stat transcription factors (Figure 1). Jak2 activation also leads to the activation of a signaling cascade through effector pathways including Ras/MAP Kinase and PI3K/Akt/mTOR, which drive cell growth. *JAK2* mutations, specifically *V617F*, are common in myelofibrosis (35–57%), ubiquitous in polycythemia vera, occurring in 96–99% of patients, and are also the most commonly mutated gene in essential thrombocythemia (50–60%) [11,12,13,14,15,16,17].

The majority of *JAK2* mutations identified in myelofibrosis are *V617F*. The JH2 pseudokinase domain of Jak2 protein has a negative regulatory effect on the JH1 kinase domain and maintains inactive Jak2 conformation [18]. Upon *V617F* mutation, however, this inhibitory effect is lost, resulting in constitutively active Jak2. The early reporting of *JAK2V617F* mutational presence in patients with myelofibrosis was through a series of publications in 2005, which ranged in number of evaluated patients (16–46) but generally identified a propensity for the mutation (35–57%) [19,20,21]. Murine models of mutant *JAK2V617F* have supported these clinical findings [16,17]. *JAK2V617F* allele burden can also be used in MF prognostication. In one 2017 model, allele burden of >50% is correlated with the median overall survival of 80 months compared with 50 months for values <50%.

Mutations in *JAK2* Exon 12 have also been identified in MPNs but are more common in polycythemia vera. Exon 12 resides between Jak2 domains JH2 and SH2 and codes for the amino acids from 505 to 547; mutations in this exon are present in approximately 3% of patients with polycythemia vera. Disease in patients with exon 12 mutations is characterized more frequently by isolated erythrocytosis rather than hyperplasia across all three lineages [22]. Its presence in myelofibrosis, however, is uncommon and therefore its prognostic impact has not been approximated in clinical studies.

### 2.2. MPL

*MPL* mutations, while much less common than *JAK2*, are enriched in a subset of patients with MF and appear to drive the disease phenotype at least partially. *MPL* is a tyrosine kinase receptor encoding for thrombopoietin (TPO) receptor, which serves as a hematopoietic stem cell (HSC) growth factor receptor (Figure 1). *MPL* is normally activated by binding of its ligand TPO. *MPL* mutations lead to constitutively active Mpl which turns on the downstream JAK pathways, prompting both HSC renewal and megakaryocyte development [23]. Its presence in myelofibrosis has varied across reported studies from as low as non-existent to up to 7% of patients [24]. Mutations in polycythemia vera and essential thrombocythemia are even less common and, respectively, have only been reported in up to 1% and 5% of patients in each disease state [13,14,24,25].

The role of mutated *MPL* in myelofibrosis first came to prominence in 2006 when Levine and colleagues performed exomal sequencing of the trans- and juxtamembrane domains of EPOR, GCSFR, and Mpl in comparison with germline DNA. Results were most notable for an activating mutation in the *MPL* (*W515L*) transmembrane domain in 4/45 (9%) patients with *JAK2*-negative myelofibrosis. A murine bone marrow transplant model with *MPLW515L* expression also induced thrombocytosis, reticulin fibrosis and splenomegaly, a phenotype not observed with wildtype *MPL* expression [26]. The estimated median overall survival in patients with *MPL* mutations in myelofibrosis is 221 months [17].

### 2.3. CALR

*CALR* mutations in myelofibrosis vary in reported frequency (25–53%) but are more common than in patients with PV (not reported) or ET (25–27%) [12,13,14,24,27,28]. The exact prognostic significance of mutated *CALR* in MF is under investigation but its presence has been correlated with increased overall survival in the absence of accompanying *JAK2* mutations [29].

The *CALR* gene, located on chromosome 19’s short arm, contains 9 exons and encodes for calreticulin, which is a chaperone protein in the endoplasmic reticulum and functions to maintain calcium homeostasis and protein folding [30,31]. The identification of somatic mutations of *CALR* in MF came in the form of two primary studies, one of which was published in 2013 by Klampfl and authors, who observed that an estimated 30–45% of patients with either primary myelofibrosis (PMF) or essential thrombocythemia (ET) had no previously known identifiable clonal mutations, including *JAK2* and *MPL* mutations [29]. Authors then performed whole-exome sequencing in six patients diagnosed with PMF without mutations in *JAK2* or *MPL* and identified *CALR* mutations in all sequenced patients. An expanded screening of 1107 samples from patients with various MPNs identified *CALR* mutations in 88% of patients with PMF when *JAK2* and *MPL* mutations were absent and confirmed that such patients had longer overall survival (OS) and lower risk of thromboses than those with *JAK* mutations.

A second study from the same year (and journal) further expanded sequencing analyses, including Sanger, whole-exome, and whole-genome sequencing, in 1397 patient samples, collectively identifying 32 patients with primary MF, 18/32 (56%) of whom were *CALR* mutated [32]. These findings, combined with those reported by Klampfl et al. strengthened the assertion that somatic mutations in *CALR* are common and are likely driver mutations in primary myelofibrosis in the absence of *JAK2* and *MPL*. Frameshift mutations in *CALR* exon 9, in a separate study, were associated with a median overall survival of 131 months in patients with primary myelofibrosis [17]. Lastly, murine models of *CALR*, including a *CALR*^del52^ conditional inducible knock-in mutation in an Mx-Cre model, have again yielded similar findings and revealed progression from essential thrombocythemia (ET) to MF in homozygous mutant mice [33].

It is also essential to understand the cumulative relationship between these classic driver mutations, which was explored by Araki and authors in a 2016 study published in *Blood* [34]. Here, the authors reported that in UT-7/TPO cells, Jak2-bound c-Mpl preferentially associated with mutant *CALR* compared with wildtype. Mutant *CALR* was also found to induce Jak2 phosphorylation and activation of Jak2 downstream signaling, an effect that can be blocked by Jak2 inhibitors. Furthermore, in hematopoietic stem cells harboring *CALR* mutations, c-Mpl was required for TPO-independent megakaryopoiesis. Although the mechanism by which CALR mutations lead to activation of JAK signaling remains to be fully elucidated, it can be concluded from this study and related reports that mutant *CALR* activates signaling molecules downstream of Jak2 via association with c-Mpl and ultimately drives the development of MPNs. A depiction summarizing the interconnectivity between these driver mutations is included in Figure 1.

## 3. Triple-Negative Myelofibrosis

Triple-negative myelofibrosis (TN MF) is diagnosed in the absence of *JAK2*, *MPL*, and *CALR* mutations while otherwise meeting morphologic and laboratory criteria for myelofibrosis. One 2021 study reported on a single-center experience of 626 patients with MF over an 18-year period and identified triple-negative disease in 38 (6%) patients [39]. Higher risk disease by both the dynamic and genetically inspired international prognostic scoring systems was observed more frequently in TN MF than in non-TN disease (65.8 vs. 58.1% DIPSS, 62.5 vs. 47.1% GIPSS). Median overall survival was also shortened in TN compared with *JAK2-, MPL-*, or *CALR-*mutated diseases at 37.4 vs. 85.7 months. Interestingly, mutations in *CBL*, *IDH2*, *GNAS, SETBP1*, and *SRSF2* were enriched in triple negative disease. A working theory in myelofibrosis is that the acquisition of additional somatic mutations drives a more high-risk phenotype and is associated with disease advancement. In the era of expanded molecular analyses early in the diagnosis of MF, reports on the impact of these mutations are growing in the MF literature. The mutations described in the following sub-sections commonly co-exist with *JAK2*, *MPL*, and *CALR* mutations in patients with myelofibrosis and, while they fall within broad categories (such as proteins affecting splicing and epigenetic regulation), each has its own unique mechanism. They are also enriched in patients with triple-negative disease. The impact of several of these mutations, specifically *ASXL1*, *EZH2*, *IDH1/2*, and *SRSF2*, directly enhances a patient’s molecular risk stratification. In the MIPSS70v2 system, a patient is assigned 2 points if one of these high molecular risk (HMR) mutations is present and 3 points if ≥2 HMR’s are present [7].

One proposed mechanism behind this risk enhancement is that more mutations create more disruption to intracellular signaling. Another theory is that when multiple clones are driving the disease, some respond to therapy while others are persistent and become more dominant drivers of myeloproliferation. A representative summary of the various mechanisms of these additional somatic mutations as well as their presence as well as impact in various myeloproliferative neoplasms as compared to classical driver mutations is summarized in Table 1.

## 4. Mutations in Epigenetic Regulators

As in other myeloid neoplasms, epigenetic regulators that modify chromatin structure to regulate gene expression play an important role in the pathogenesis of MF. Mutations of *ASXL1* and EZH2, for instance, have identified in myelofibrosis. The presence of these mutations is often associated with an overall poorer outcome and therefore provides prognostic value in disease. By comparison with other myeloproliferative neoplasms in PV and ET as depicted in Table 1, both *ASXL1* and *EZH2* mutations are more common in myelofibrosis.

### 4.1. ASXL1

*ASXL1* is an epigenetic regulator of genes involved in chromatin remodeling, specifically a polycomb repressive complex protein (PRC), deletion of which has been implicated in impaired hematopoiesis and acceleration of myeloid neoplasms [62]. Guglielmelli, Coltro, and investigators reported in 2022 an analysis of 523 patients with MF at various disease stages, including primary myelofibrosis (PMF), pre-MF, and post-polycythemia vera (PPV-MF), in which *ASXL1* mutations were identified in 157 (30%) of patients [63]. *ASXL1* mutations in PMF were associated with several phenotypic characteristics of higher-risk disease, including leukocytosis, lower hemoglobin and platelets, peripheral blast presence, and marrow fibrosis grade ≥ 2. Amongst patients formally diagnosed with high-risk disease, *ASXL1*^mut^ were identified in 62% of patients and were associated with a shortened median overall survival compared with *ASXL1*^WT^ at 47 and 102 months, respectively (*p* = 0.0240).

### 4.2. EZH2

*EZH2*, in mammals, is a subunit of the larger histone methyltransferase polycomb repressive complex 2 (PRC2) and is felt to play a role in the progression of various tumors [64]. Like *ASXL1*, it was also explored by Guglielmelli in 2011 in an analysis of 370 patients with primary myelofibrosis and 148 with post-PV/post-ET MF, where genotyping for *EZH2* identified mutations in 5.9%, 1.2%, and 9.4% of patients with primary MF, post-PV MF and post-ET MF, respectively [65]. Clinically, elevations in peripheral blasts, leukocytes and splenomegaly were significantly higher in *EZH2*-mutated PMF. *EZH2* mutations in PMF were also associated with significantly decreased leukemia-free and overall survival.

## 5. Mutations in Splicing Factors

Splicing factors are essential to pre-mRNA regulation in normal cellular functions. Mutations of two such factors in *SRSF2* and *U2AF1* have been identified as high molecular risk mutations in myelofibrosis as both are associated with lower median overall survival compared with wildtype [49,66,67]. Both *SRSF2* and *U2AF1* mutations are rare in polycythemia vera (both < 2%) and essential thrombocythemia (collectively 1–3% as shown in Table 1) [13,15].

### 5.1. SRSF2

Serine and arginine-rich splicing factor 2 (*SRSF2*) is multifaceted but appears to be an essential component of pre-mRNA alternating splicing machinery, mutations of which have been identified in approximately 6–17% of patients with myelofibrosis [46,47,68]. A recent investigation into the prognostic significance of *SRSF2* in 187 patients with PMF identified significantly lower median overall survival in patients harboring *SRSF2* mutations (24 months) compared with *SRSF2* wildtype (65 months); 2-year leukemia risk was also higher with mutated *SRSF2* (30 vs. 8%) [47]. Other significant associations with *SRSF2* mutations included co-mutated *IDH1*, higher risk stratification by dynamic international prognostic scoring system (DIPSS) and advanced age.

### 5.2. U2AF1

*U2AF1* is also a splicing factor which encodes RNA-binding protein and is involved in recognizing the 3′ splice site facilitating U2 snRNP recruitment in the process of pre-mRNA splicing; it was also recently reported to promote survival of hematopoietic stem/progenitor cells (HSPCs) [69]. Tefferi and others in 2018 examined 491 patients with PMF and identified *U2AF1* mutations in 77 (16%) patients, 50 of which involved the *Q157* variant and 26 involving the *S34* or other variants [48]. Median overall survival was 2.9 years for patients with *U2AF1*, variant *Q157*, significantly shorter than patients with the *S34* variants and unmutated *U2AF1* (5.8 and 5.5 years, respectively). *U2AF1 Q157* was also identified to independently contribute to prognosis in PMF by multivariate analysis and is now included in the MIPSS70+ version 2.0+ risk assessment tool [70].

## 6. Mutations in IDH-Regulators of Cellular Metabolism

Isocitrate dehydrogenase (*IDH*) mutations are relatively uncommon in patients with myelofibrosis but, when present, appear to be associated with lower overall survival compared with wildtype [50]. *IDH* mutations in PV and ET are even more rare, both occurring in <2% of patients [13,15]. Isocitrate dehydrogenase (*IDH*) normally catalyzes the conversion of isocitrate to alpha-ketoglutarate and in mammals, both isoenzymes IDH1 (cytosolic) and IDH2 (mitochondrial) are NADP-dependent [66,71]. Mutant *IDH1/2* in leukemic cells instead yields a separate oncometabolite called 2-hydroxyglutarate (2-HG), leading to multiple effects that include hematopoietic differentiation and inhibition of the TET methylcytosine dioxygenases, which are instrumental in DNA demethylation [67]. *IDH* mutations have been identified (and are targeted) in numerous solid and hematologic neoplasms, including acute myeloid leukemia, cholangiocarcinoma, and glioma [72]. The presence and impact of *IDH* mutations in PMF was explored by Tefferi in an analysis of 301 patients with PMF, 12 of whom (4%) were identified to have the mutation (5 *IDH1*, 7 *IDH2*) [50]. Inferior overall survival (OS) was observed in multivariate analysis in *IDH*-mutated patients. Interestingly, 6 of these 12 patients also possessed *JAK2*V617 mutations, and a more pronounced impact on LFS and OS was observed in patients co-mutated with *IDH* and *JAK2*, which authors suggested may represent “leukemogenic collaboration” between the two.

Regarding the impact of *IDH* mutations on therapy, there have been no studies examining IDH 1 nor 2 inhibitors as monotherapy in MF, although there was a Phase II trial recently completed in March 2023 evaluating combination of ruxolitinib + enasidenib in patients with *IDH2* mutations and either blast-phase MPNs or chronic myelofibrosis [73]. Results are forthcoming but further investigation into the role of IDH inhibitors in myelofibrosis beyond this study is warranted to continue expanding available therapies.

## 7. Mutations in TP53 Tumor Suppressor Gene

*TP53* mutations vary by prevalence in myelofibrosis (1–13%) but are associated with both increased risk for transformation to leukemia and poorer overall survival when present [52,53]. Similar to mutations of epigenetic, splicing, and cellular metabolism regulators, *TP53* mutations are comparatively less common in polycythemia vera (<2%) and essential thrombocythemia (1–4%) [13,15,45].

*TP53* is a tumor suppressor gene whose wildtype function is well-established in cell cycle control, DNA damage repair, and apoptosis and whose mutated forms, both inherited (germline) or acquired (somatic), have been implicated in the pathogenesis of numerous hematologic and visceral neoplasms [51,74]. *TP53* has been recently evaluated in a multicenter cohort of 349 patients with myelofibrosis, 13% (49) of whom were *TP53*-mutated, with a median variant allele frequency (VAF) of 50% [52]. Authors also evaluated for multi-hit *TP53* configurations and identified 30 of the 49 patients (8.6% of the entire cohort) possessing multi-hit mutations of *TP53*. The outcome data revealed a marked difference between median survival in *TP53*-mutated (1.5 years) and *TP53* wildtype (13.5 years) patients (*p* < 0.001). A similar trend was reported in leukemic transformation, seen in 20% versus 2% of *TP53* mutant and wildtype patients, respectively (*p*-value again <0.001).

**Figure 2:**
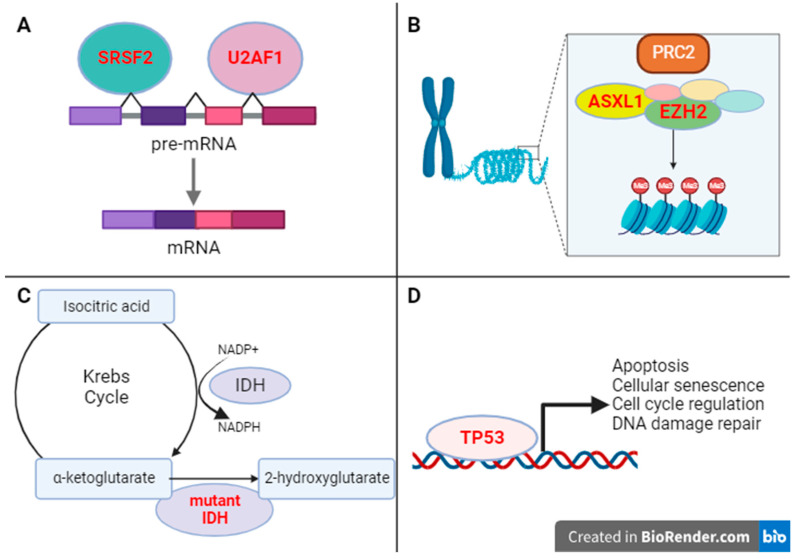
Functions of mutations other than JAK/MPL/CALR commonly found in MF. (**A**) Loss-of-function mutations in splicing factors *SRSF2* and *U2AF1* lead to altered mRNA splicing in myelofibrosis [49,66]. (**B**) Loss-of-function mutations in *ASXL1* and *EZH2* lead to impaired epigenetic regulation and are resultantly associated with high-risk MF disease features, including elevation of blasts in the peripheral blood and shortened overall survival [65,70]. (**C**) *IDH 1/2* mutations have been studied in myeloid neoplasms and other malignancies, and when present, yield an oncometabolite termed 2-hyroxyglutarate and are correlated with inferior overall survival in myelofibrosis [73]. (**D**) *TP53* loss-of-function mutations, lastly, are also identified in MF and are associated with both shortened median overall survival and increased risk for leukemic transformation [53].

Taken together, while mutations that lead to hyperactivation of JAK/STAT signaling, mostly in *JAK*/*CALR*/*MPL*, are highly prevalent in both PV/ET and MF, MF patients often acquire additional mutations in genes encoding epigenetic regulators (*ASXL1* and *EZH2*), splicing factors (*SRSF2* and *U2AF1*), metabolic enzymes *IDH1/2*, and *TP53* tumor suppressor gene. These additional mutations are often associated with poor outcome and provide important prognostic information in MF patients (Table 1 and Figure 2). Strategies targeting these mutations or mutated pathways may lead to improved response in MF and remain to be investigated.

## 8. Expanding the Molecular Horizon in Myelofibrosis

While additional mutations and/or signaling pathways implicated in the pathogenesis of both marrow fibrosis and HSPC proliferation are expanding our present understanding of molecular landscape of this myeloproliferative neoplasm, treatment for MF remains limited and resistance to Jak inhibitors is a common theme in clinical practice and is a likely driver of worsening disease. Effort has been focused on identifying mechanisms of resistance to Jak inhibitor therapy and developing strategies to overcome this resistance. One of the proposed mechanisms for this resistance in pre-clinical models is up-regulation in Ras signaling, and multiple related pathways are actively being explored preclinically to better characterize the relationship between Ras signaling and myelofibrosis pathogenesis.

Y-box binding protein (YBX1) is a multifaceted protein that binds to nucleic acid, with functions in transcription as well as mRNA splicing. It is proposed to regulate various genes associated with cancer resistance. YBX1 phosphorylation also leads to nuclear localization and activation of YBX1 target genes, which in a recent study was increased in *JAK2V617F* cells in an MEK/ERK-dependent manner [75]. In an RNA interference screen targeting proteins enriched in JAK2V617F cells compared with JAK2 wild type cells, a loss of YBX1 sensitized cells to Ruxolitinib-mediated apoptosis [76]. Pharmacologic inhibition of Jak combined with YBX1 inactivation also induced apoptosis in Jak-2-dependent human and mouse cells. Authors concluded that co-targeting YBX1 and Jak2 can be an effective strategy in eradicating *JAK2*-mutant hematopoietic cells in MPNs. This study also provides evidence that MEK/ERK signaling pathway may be involved in resistance to JAK inhibitors.

In addition, upstream cytokine signaling that activates MEK/ERK has been shown to carry potential for therapeutic targeting in myelofibrosis. One of the recent studies in this space published in Blood by Melo-Cardenas and others developed various models of MF to analyze cytokine signaling [77]. Their first set of experiments to this goal was an analysis of cytokine profiles in mice before and after fibrotic marrow development, which was coupled with single-cell RNA sequencing of marrow populations. Here, IL-13 was elevated in the marrow of mice with MF, which induced both collagen biosynthesis and transforming growth factor beta (TGFβ) surface expression and promoted mutant megakaryocytic growth. In a correlative study with samples from patients with MF, IL-13 levels were increased in plasma and the expression of its receptor was increased in marrow megakaryocytes. Authors ultimately theorized through these and related experiments that IL-13 was not only contributory to MF progression but also a potential target in developing MF-directed therapies.

On this note, cytokine signaling is implicated not only in the pathogenesis of myelofibrosis but also the downstream activation of Ras/MAP Kinase signaling, which has led to numerous clinical and pre-clinical evaluations as to the role of Ras/MAP Kinase in the disease state. It is worth noting that while it is well-known that Jak/Stat can act as an upstream regulator of Ras signaling, cytokines can also activate Ras via alternative tyrosine kinase receptor pathways.

## 9. Ras/MAP Kinase Signaling in Myelofibrosis

Ras/MAP Kinase signaling begins when a ligand (cytokine or growth factor) binds to a receptor tyrosine kinase, leading to receptor dimerization and autophosphorylation [78,79]. Various adaptor proteins, including GRB2 and SOS, then bind to the now-activated receptors and recruit guanine nucleotide exchange factors (GEFs), which facilitate the exchange on Ras of guanine diphosphate (GDP) for guanine triphosphate (GTP) and activates Ras proteins. GTP-bound Ras can then interact with the Raf kinase, leading to the sequential activation and phosphorylation of MEK and then ERK, a process better known as the MAP kinase cascade. Key regulators in this process are neurofibromin 1 (NF1), which as depicted in Figure 1 inactivates Ras-GTP by stimulating GTPase, and SHP2 (encoded by *PTPN11*), a Ras-activating tyrosine phosphatase [36,59]. Mutations in various proteins either directly or indirectly involved in this process can have various impacts on Ras activation. CBL, for instance, is a ubiquitin ligase that degrades receptor tyrosine kinase and when mutated leads to a loss of function of CBL and increased Ras activation [80]. Ras/MAP Kinase pathway signaling is also theorized in various models to be interconnected with a downstream effector PI3K/Akt/mTOR pathway, collectively then enacting various cellular processes that regulate cell growth and survival [81]. The relationship between Jak-Stat and Ras signaling is under investigation but Jak-Stat is likely contributory at least to an extent to Ras activation [82].

Despite the available therapies targeting Jak-Stat signaling, myelofibrosis frequently worsens or transforms in the presence of the high-risk molecular mutations, particularly those in the Ras/MAP Kinase pathway. A few recent studies investigated the role of Ras pathway mutations in MF. Coltro and investigators in 2020 examined 464 patients with MF, 59 of whom had *RAS*/*CBL* mutations (5.4% *NRAS*, 2.8% *KRAS*, and 5.6% *CBL*) [57]. These mutations were associated with inferior overall survival and a higher 5-year incidence of leukemic transformation. These investigators also reported that, of the 61 patients treated with Jak inhibitors, response in spleen size and symptoms were lower in *RAS*/*CBL* mutant cohort, which was confirmed on logistic regression analysis. A similar 2020 study reported next generation sequencing data of 16 target genes in 723 patients with either primary or secondary MF and identified subclonal *N/KRAS* mutations in 6% of patients [8]. *RAS* mutations were also associated with advanced MF features, including leukocytosis and high burden of high-risk mutations; *RAS*-mutated patients also had shortened 3-year overall survival.

The frequency of Ras pathway mutations in JAK inhibitor resistant MF patients was evaluated in a recent study which retrospectively evaluated 113 patients with MF with baseline molecular data, focusing specifically on patterns in Jak inhibitor failure [83]. Amongst 49 patients with new comparative molecular data available who had failed Jak inhibitors, 29 different emergent mutations were identified in 19 patients (39%), 9 of which (18%) were within the Ras/MAP Kinase pathway.

More recently, a study utilizing Mi-Oncoseq analysis investigated Ras pathway mutations in in 216 patients with either primary myelofibrosis or ET/PV pre-primary myelofibrosis [55]. Here, mutations in the Ras/MAP Kinase pathway, including *K/NRAS*, *MAPK*, *CBL*, and *NF1* were present in up to 25% of patients and were associated with significantly higher platelet and leukocyte counts (*p*-valves of 0.035 and 0.015, respectively). Interestingly, *KRAS* and *NRAS* mutations were exclusive to the MF cohort.

In summary, there is emerging evidence that mutations activating Ras signaling pathways are frequently found in MF but not earlier stage pre-myelofibrosis such as PV and ET patients. These mutations likely confer a proliferative and high-risk phenotype. More importantly, Ras pathways mutations are enriched in MF patients who fail JAK inhibitor therapies, raising the possibility that hyperactive Ras signaling is a potential mechanism of resistance to JAK inhibitors. Treatment targeting the Ras/MAPK pathway therefore can provide a strategy to overcome JAK inhibitor resistance. A concise summary of commonly utilized Jak inhibitors in myelofibrosis and various small molecule inhibitors in the MAP Kinase pathway are summarized in Table 2.

## 10. Targeting Ras Signaling in Myeloid Neoplasms

Various studies have directly evaluated the effect of inhibiting individual components of Ras signaling pathway, specifically through Mek or Erk 1/2 inhibitors, as an attempt to overcome therapeutic resistance in myeloid neoplasms. Most of these studies were conducted in AML with disappointing results from monotherapies. One example was a Phase II study evaluating binimetinib in 19 patients with either relapsed/refractory acute myeloid leukemia (AML) or myelodysplastic syndrome (MDS), 14 of whom harbored *RAS* mutations [92]. Ultimately, only 13 patients were evaluable and 12 (92%) did not achieve complete response (CR); authors suggested combining such inhibitors with other therapies for future studies. Another Phase II trial evaluated the oral Mek 1/2 inhibitor selumetinib in 47 patients who were either ≥60 with untreated AML or any age with relapsed/refractory AML [93]. Responses were absent in the *FLT3 ITD* and *NRAS*-mutated cohorts and the only patient with a *KRAS* mutation had a minor response (which itself was unconfirmed).

These limited results with individual agents have prompted investigations into combination therapies in myeloid diseases. Trametinib, an oral Mek 1/2 inhibitor, for example, was examined in a Phase II study in combination with an Akt inhibitor in 23 patients with *RAS*-mutant AML, none of whom achieved CR [94]. Pre-clinical investigations, however, are expanding on combinational regimens. One study published in Nature Communications, for instance, argued that osteopontin (or ‘OPN’) promoted the proliferation of mesenchymal stromal cells, collagen production, and marrow fibrosis induced by TPO receptor agonist (romiplostim) [95]. Inhibition of ERK 1/2 was then observed to substantially reduce the production of OPN and, in turn, marrow fibrosis in vivo. Recognizing that Ras/MAP Kinase inhibition alone is likely inadequate, Ross and investigators developed a multi-kinase inhibitor targeting Mek and P13K termed LP-182, which was designed for preferential lymphatic absorption, and were successful in ameliorating the myelofibrosis phenotype as well as improving overall survival in their animal models [96].

Another strategy that is in line with the concept of synergistic targeting but is more relevant to the known existing therapeutic landscape in myelofibrosis is combining Mek (or other agents targeting MAP Kinase pathway signaling) with Jak inhibitors to manage resistance to Jak inhibitor monotherapies. Pandey and investigators in 2022, for instance, further investigated mRNA splicing alterations and identified intron retention in mRNAs encoding for Mek/Erk signaling. One observation was that the expression of MAPK-interactive kinase 1 (MNK1) was diminished in YBX1-depleted *JAK2*-V617F cells and was required for Erk signaling [75]. Moreover, MNK1 inhibition enhanced Jak2 inhibitor-mediated cell death and, together with Mek inhibition, induced apoptosis of CD34+ *JAK*-V617 cells. A correlative in vivo study in PDX mouse models using transplanted *JAK*-V617F-expressing marrow from patients with MPN compared the effects of trametinib + ruxolitinib to ruxolitinib alone [76]. Authors reported significant growth inhibition as well as molecular remission of transplanted cells in the ruxotlinib + trametinib group compared with ruxolitinib alone, suggesting a synergy between ruxolitinib and Ras/MAP kinase pathway inhibitors.

The observation that Mek/Erk targeting increases susceptibility of MPNs to JAK inhibitors has been explored by other groups as well. Stivala and investigators in 2019 reported in vitro suppression of Mek/Erk activation with Type I and II Jak2 inhibition in MPN cell lines and in an ex vivo model [97]. This suppression, however, was lost in vivo in *MPL*W515L and *JAK2*V617F mice after Jak2 inhibition alone but combined Mek/Jak inhibition was able to suppress activation of Mek/Erk. Binimetinib plus ruxolitinib exposure for 2 weeks was also superior to ruxolitinib alone in the reduction in reticulin fibrosis, hepatosplenomegaly and megakaryocytic hyperplasia in the bone marrow. The difference between ex vivo and in vivo MAPK activation is at least partially explained by the cytokines that the mutant cells are exposed to in vivo. PDGFRα notably remained activated and production of PDGF-AA/PDGF-BB (which are the subunits of PDGFR) persisted following Jak2 inhibition in vivo and PDGF-BB, on exposure to ruxolitinib, maintained activation of Erk [98]. This observation was consistent with the known function of PDGF-BB serving as a bypass for Erk activation, leading to diminished efficacy of Jak2 inhibition, and supported the utility for dual MAP Kinase pathway/Jak2 inhibition in the management of MPNs.

These studies collectively suggest that *RAS* mutations and Ras signaling activating mutations are frequently detected in MF and are associated with an adverse outcome. In addition, Ras signaling is activated despite mutational status or Jak inhibition, possibly through increased cytokine signaling in MF, raising the possibility that Ras pathway targeting strategies may offer therapeutic benefit to MF patients.

## 11. Conclusions and Future Directions

The pathogenesis of primary myelofibrosis is not determined solely by alterations in *JAK2*, *CALR*, and *MPL* and likely involves a much broader landscape of somatic mutations, which themselves affect both clinical features of disease and overall survival in various capacities. Of these, Ras/MAP Kinase pathway mutations represent an area of growing clinical interest, both mechanistically and as potential therapeutic targets in myelofibrosis. Expanded translational analyses and early phase clinical trials exploring the role of Ras/MAP Kinase signaling and manipulation are needed to further targeted therapies in this myeloproliferative neoplasm.

## Figures and Tables

**Table 1:** Mutations implicated in the pathogenesis and/or molecular characterization of myelofibrosis (MF), polycythemia vera (PV), and essential thrombocythemia (ET). “Triple negative” refers to the designation of *JAK2*, *CALR*, and *MPL* wildtype myelofibrosis. The references for each incidence listed above are denoted by brackets.

Mutation	Normal Function	Incidence in MF (%)	Incidence in PV (%)	Incidence in ET (%)
**Driver mutations in myeloproliferative neoplasms**				
*JAK2*	Nonreceptor tyrosine kinase mediating normal hematopoiesis, cell growth [19,20,21]	35–57 [19,20,21]	96–99 [11,12]	50–60 [13,14,40]
*MPL*	Protooncogene encoding for thrombopoietin receptor (TPOR) [23]	0–7 [24,27]	0–1 [24,25]	5 [13,14]
*CALR*	Chaperone ER protein maintain calcium homeostasis and protein folding [31,32]	25–53 [27,28]	0 [12,24]	25–27 [13,14]
“Triple negative”	-	10–15 [41]	Rare	35–52 [15]
**Mutations in Regulators of Epigenetic Regulators**				
*ASXL1*	Epigenetic regulator of genes involved in chromatin remodeling [42]	13–38 [42]	8.2 [43]	7–20 [13]
*EZH2*	Epigenetic regulator of posttranslational histone modification [44]	6–13 [44]	0–3 [45]	2–4 [13,15]
**Mutations in Regulators of Splicing Factors**				
*SRSF2*	Splicing of pre-mRNA [46]	3–17 [46,47]	<2 [15]	2–3 [13]
*U2AF1*	Splicing factor involved in pre-mRNA splicing, promotion of HSPC survival [48]	16–22 [48,49]	<2 [15]	1 [13]
**Mutations in Regulators of Cellular Metabolism**				
*IDH*	Catalyzation of isocitrate decarboxylation to yield 2-oxoglutarate (2-OG) [50]	4 [50]	<2 [15]	1 [13]
**Mutations in Tumor Suppressor Genes**				
*TP53*	Tumor suppression, cell cycle regulation [51]	1–13 [52,53]	<2 [15]	1–4 [13,45]
**Ras/MAP Kinase Pathway Mutations**				
*KRAS/NRAS*	Proto-oncogene with GTPase activity; K/Nras are two different Ras isoforms [8,54]	2–15/4.4–14 [8,55]	<2 (*NRAS*) [15]	<2 (*NRAS*) [15]
*CBL*	E3 ubiquitin ligase; negatively regulates various receptor tyrosine kinases [56]	5–13 [55,57,58]	<2 [15]	1–2 [13]
*NF1*	Inactivation of Ras-GTP through stimulation of constitutional GTPase [59]	0–6 [55,58]	Rare	Rare
*BRAF*	Cytosolic serine/threonine kinase regulating MEK, ERK [60]	0–1 [61]	Rare	Rare
*PTPN11 (SHP2)*	PTPN11 encodes for SHP2, a tyrosine phosphatase activating Ras/MAP Kinase [36]	1 [8]	<2 [15]	<2 [15]

**Table 2:** JAK and Ras/map Kinase Signaling inhibitors common used as cancer therapies. Abbreviations: GVHD: graft-versus-host disease; NSCLC: non-small cell lung cancer; MF: myelofibrosis. References are denoted by brackets.

Inhibitor	Mechanism/Targets	FDA-Approved Treatment Indications
Ruxolitinib	Jak1; Jak2 [84]	Proliferative myelofibrosis; Glucocorticoid-refractory GVHD [38,85]
Fedratinib	Jak2; FLT3; BRD4 [38]	Symptomatic, severe cytopenic MF [38]
Pacritinib	Jak2; IRAK1 [38]	Proliferative and moderately cytopenic MF [38]
Dabrafenib	BRAF, CRAF [86]	BRAF V600E-mutated NSCLC; melanoma [87]
Vemurafenib	BRAF [86]	BRAF V600E-mutated NSCLC; melanoma [87]
Cobimetinib	Mek 1/2 [88]	Melanoma; histiocytic disorders [87,89]
Trametinib	Mek 1/2 [90]	Melanoma; histiocytic disorders [87,91]

## Data Availability

No new data were created or analyzed in this study. Data sharing is not applicable to this article.
